# Microbiota, Tryptophan and Aryl Hydrocarbon Receptors as the Target Triad in Parkinson’s Disease—A Narrative Review

**DOI:** 10.3390/ijms25052915

**Published:** 2024-03-02

**Authors:** Paulina Iwaniak, Maja Owe-Larsson, Ewa M. Urbańska

**Affiliations:** 1 Department of Experimental and Clinical Pharmacology, Medical University of Lublin, 20-059 Lublin, Poland; paulina.iwaniak@umlub.pl; 2 Department of Histology and Embryology, Center of Biostructure Research, Medical University of Warsaw, Chałubińskiego 5, 02-004 Warsaw, Poland; maja.owelarsson@onet.pl; 3 Laboratory of Center for Preclinical Research, Department of Experimental and Clinical Physiology, Medical University of Warsaw, Banacha 1B, 02-097 Warsaw, Poland

**Keywords:** Parkinson’s disease, microbial–intestinal–brain axis, diet, aryl hydrocarbon receptor, microbiota, aging, tryptophan, kynurenine, probiotics, individualized therapy

## Abstract

In the era of a steadily increasing lifespan, neurodegenerative diseases among the elderly present a significant therapeutic and socio-economic challenge. A properly balanced diet and microbiome diversity have been receiving increasing attention as targets for therapeutic interventions in neurodegeneration. Microbiota may affect cognitive function, neuronal survival and death, and gut dysbiosis was identified in Parkinson’s disease (PD). Tryptophan (Trp), an essential amino acid, is degraded by microbiota and hosts numerous compounds with immune- and neuromodulating properties. This broad narrative review presents data supporting the concept that microbiota, the Trp-kynurenine (KYN) pathway and aryl hydrocarbon receptors (AhRs) form a triad involved in PD. A disturbed gut–brain axis allows the bidirectional spread of pro-inflammatory molecules and α-synuclein, which may contribute to the development/progression of the disease. We suggest that the peripheral levels of kynurenines and AhR ligands are strongly linked to the Trp metabolism in the gut and should be studied together with the composition of the microbiota. Such an approach can clearly delineate the sub-populations of PD patients manifesting with a disturbed microbiota–Trp-KYN–brain triad, who would benefit from modifications in the Trp metabolism. Analyses of the microbiome, Trp-KYN pathway metabolites and AhR signaling may shed light on the mechanisms of intestinal distress and identify new targets for the diagnosis and treatment in early-stage PD. Therapeutic interventions based on the combination of a well-defined food regimen, Trp and probiotics seem of potential benefit and require further experimental and clinical research.

## 1. Introduction

The high prevalence of neurodegenerative diseases in the elderly population presents an important therapeutic and socio-economic challenge. The process of aging is determined by a variety of factors, including non-modifiable aspects, such as genetic predisposition, and modifiable aspects, such as, environmental influence, individual lifestyle, physical activity and eating habits. In recent years, a properly balanced diet and microbiome diversity have received increasing attention as targets for therapeutic interventions in neurodegeneration.

Parkinson’s disease (PD) is the second most common progressive neurodegenerative disorder of aging. Neuropathological features involve the loss of dopaminergic neurons in the substantia nigra (SN) and the intraneuronal formation of Lewy bodies, aggregates built from α-synuclein, neurofilaments and ubiquitin [1]. Clinically, PD manifests as motor symptoms comprising resting tremors, bradykinesia, akinesia, rigidity and gait disturbance. Anxiety, depression and cognitive impairment follow, ultimately leading to severe disability [2].

Despite immense scientific efforts, the etiology of PD is not fully elucidated, yet a number of genetic and environmental risk factors have been identified [3]. Hereditary PD accounts for approx. 5% of cases. In one-fifth to one-third of inherited PD, more than 90 independent genetic risk signals in 37 loci were detected [4]. An increased probability of developing PD has also been linked with traumatic brain injury, dairy and alcohol consumption, diabetes, high cholesterol, hypertension and exposure to pesticides [5,6]. On the other hand, physical exercise, the intake of caffeine, the use of non-steroidal anti-inflammatory drugs and special diets, such as Mediterranean or so-called Mediterranean-DASH Intervention for Neurodegenerative Delay (MIND) diets were shown to lower PD risk [7]. Cellular and molecular pathways leading to neuronal loss are complex and involve, among others, mitochondrial dysfunction, impaired regulation of the proteome, excitotoxicity or neuroinflammation. Since the pathogenesis of PD has been the subject of a number of timely reviews recently (see, for example, [8,9,10]), it will not be presented here in detail. 

The standard therapy for PD is symptomatic and based on the enhancement of dopaminergic transmission in the basal ganglia. The oral administration of a dopamine precursor, levodopa, is initially highly effective, but prolonged therapy is associated with reduced drug efficacy and the occurrence of adverse effects [11]. Novel invasive treatments, like the deep brain stimulation of the subthalamic nucleus or globus pallidus internus, result in an improved quality of life, yet a number of serious complications may arise [12]. An early diagnosis of PD followed by optimal preventive therapy appears, therefore, to be of crucial value. 

A microbiome encompasses all living microorganisms present within a given environment, i.e., microbiota, together with their metabolites, structural fragments and the molecules produced by the host exposed to these microbiota [13]. Increasing evidence suggests that the gut microbiome may play a key role in age-related inflammation, a factor linked with neurodegeneration, aging and cognitive decline [14,15]. Two decades ago, Braak proposed that PD starts within the intestines, in the process of the retrograde transport of misfolded α-synuclein from the gastrointestinal tract to the brain [16]. The following years presented numerous clinical data revealing the existence of microbial dysbiosis among PD patients [17,18]. Furthermore, the existence of the gut–brain axis and a reciprocal connection between intestinal and brain compartments are well recognized [19]. Therefore, dietary intervention and changes in the composition of gut microbiota seem attractive ways of modulating intestinal metabolism and, in turn, the gut–brain axis. This may result in a changed synthesis, release and intestinal absorption of compounds able to influence neuronal function [20].

Tryptophan (Trp) is an essential amino acid used for protein synthesis, and it is metabolized into several biologically active compounds, including kynurenines, indoles, melatonin and serotonin [21]. The Trp-kynurenine (KYN) pathway is the major route of Trp conversion, yielding a number of immuno- and neuroactive derivatives, collectively called kynurenines [22,23]. Alterations in the function of the Trp-KYN pathway are implicated in the pathogenesis of PD, and, accordingly, manipulations of Trp metabolism may be of therapeutic benefit [24]. Some kynurenines have been identified as agonists of the aryl hydrocarbon receptor (AhR), able to activate AhR signaling in the periphery and in the brain. AhR is a transcription factor widely expressed by immunocompetent cells, and is thus involved in the modulation of the immune response [14]. Increasing evidence suggests that changes in the composition of the gut microbiota affect local Trp metabolism and, in turn, impact brain function [25,26]. Thus, dietary modifications of the Trp-KYN pathway seem to be of potential value as an easy, socially acceptable therapeutic approach able to alleviate certain neurological and psychiatric diseases, including PD [27,28]. This narrative review presents data on the correlation between gut microbiota and the availability of Trp and AhR function in PD and discusses whether dietary interventions may improve this triad and, in turn, impede the progress of PD. 

## 2. Gut–Brain Axis Correlates Microbiota with Neuroinflammation

The composition of the human microbiome is distinctive, and the differences among host individuals are considerable. Gut colonization occurs early in life, yet the environment, especially changes in diet, can profoundly impact the gut microbial population [29]. The microbiota–brain axis concept assumes that the brain and microorganisms residing within the gut communicate bi-directionally through the immune, neuroendocrine, circulatory and enteric nervous systems [30]. Commensal species within the gut are involved in the development of the central nervous system (CNS), preserving the blood–brain barrier and maintaining the proper function of the brain [15,31]. On the other hand, certain microbiota-derived antigens and metabolites can promote a “leaky gut” syndrome and elicit intestinal inflammation. It is currently acknowledged that peripheral inflammation is a potential risk factor in various neurodegenerative diseases, especially PD (for a review, see [10,32,33,34]). Reactive microgliosis within the SN, an increased number of major histocompatibility complex (MHC) II-positive cells in various brain areas and high levels of pro-inflammatory molecules are hallmarks of PD [10]. Furthermore, sustained activation of the immune system, manifested by increased levels of cytokines and chemokines, is evident in the blood and gut of PD patients [10].

### 2.1. Gut–Neuroinflammation Axis in PD

Substantial experimental data obtained in animal models of PD revealed prominent microglial activation and other inflammatory changes preceding dopaminergic neuronal loss [35]. Such changes were detected in models evoked by intracerebral administration of neurotoxin 6-hydroxydopamine (6-OHDA), systemic application of 1-methyl-4-phenyl-1,2,3,6-tetrahydropyridine (MPTP; mitochondrial toxin) or paraquat (pesticide) and oral treatment with rotenone (a naturally occurring pesticide and insecticide) [10,36].

In line with the inflammatory hypothesis, a model of PD based on the peripheral or intranigral administration of lipopolysaccharide (LPS) was developed. Inflammation causes the destruction of SN and a behavioral sequela resembling PD [37]. Furthermore, LPS was shown to trigger a consecutive increase in α-synuclein immunoreactivity, intestinal permeability and pathological accumulation of α-synuclein in the colon, in a manner similar to that observed in PD patients [38,39]. It was discovered that α-synuclein pathology can spread from the gut to the brain along the gut–brain axis, and an injection of α-synuclein into the intestinal wall may cause pathological changes in the CNS [40,41,42]. In a model of ulcerative colitis, inflammatory changes exacerbated the LPS-induced damage to the SN [43]. PD patients manifest high serum levels of zonulin and claudin-5, markers of intestinal barrier dysfunction. Increased calprotectin, a marker of intestinal inflammation, was also detected among PD patients [33]. Furthermore, in a mouse model of PD based on acute, intranigral injection of viral vectors encoding the mutated form of α-synuclein, the latter was transported from the brain to the ileum of mice. Subsequently, α-synuclein formed insoluble aggregates and impaired intestinal function [44].

Thus, accumulating data suggest that in PD, the disturbed function of the gut–brain axis may contribute to the spread of inflammatory changes, which, in turn, may affect the development and progression of the disease. Brundin and colleagues divided the course of PD into three temporal phases mediated by triggers, facilitators and aggravators [45]. Using this concept, the inflammatory state in the gut could be viewed as the trigger that initiates the neurodegenerative processes as well as a facilitator of PD. 

### 2.2. Microbiota and Microbiome in PD

In various animal models of PD, including the MPTP- or rotenone-induced destruction of SN [46,47,48], dysbiosis and alterations in the gastrointestinal tract function were observed, often before the onset of characteristic basal ganglia dysfunction [46,49]. In the rotenone PD model, changes in intestinal permeability were linked with altered composition of the gut microbiota. Higher abundances of *Lactobacillus*, *Bifidobacterium*, *Akkermansia* and *Bacteroides* spp., accompanied by the decline of *Lachnospiraceae*, *Ruminococcaceae_UCG-014*, *Turicibacter*, *Faecalibaculum* and *Clostridium* spp., were detected in mice [47]. In the MPTP model, decreased phylum *Deferribacteres*, decreased genera *Mucispirillum* and *Clostridium* and lower biodiversity of intestinal species were described [48]. Chronic stress further exacerbated intestinal barrier dysfunction, decreased the relative abundance of fecal *Lactobacillus* and increased the mucin-degrading *Akkermansia* species, as revealed in a rotenone model [50].

The altered microbiota composition in the large intestines of PD patients shows similarities to the changes observed in animal models [51,52,53,54]. However, of more than 160 human studies on the role of gut microbiota in PD, only approximately 20 were case–control [55,56]. The resulting picture is not fully clear since there are quantitative differences between various areas of the world in terms of bacterial diversity [56]. In the USA and Canada, a significant increase in Actinobacteriota (*Bifidobacterium*), Verrucomicrobiota (*Akkermansia*), Firmicutes (*Enterococcus*, *Hungatella*, *Lactobacillus*, *Oscillospira*, *Ruminococcaceae*), Bacteroidetes and Proteobacteria, with a decrease in Firmicutes *Lachnospiraceae* (*Blautia*, *Coprococcus* and *Roseburia*), was reported. In Germany, Ireland and Finland, a raised number of Verrucomicrobiota (*Akkermansia*), Firmicutes (*Lactobacillus* and *Roseburia)*, Bacteroidetes (*Barnesiellaceae*), Actinobacteriota (*Bifidobacterium*) and Thermodesulfobacteriota (*Bilophila wadsworthia*), with a decrease in *Prevotella* from Bacteroidetes, was shown. In Italy, increased Verrucomicrobiota (*Akkermansia*), Actinobacteriota (*Bifidobacteriaceae* and *Coriobacteriaceae*), Firmicutes (*Christensenellaceae* and *Lactobacillaceae*, *Enterococcaceae*, *Lactococcus*, *Oscillospira*), Proteobacteria (*Citrobacter*, *Klebsiella*, *Salmonella* and *Shigella*) and Bacteroidetes, with a reduced number of other Firmicutes (*Lachnospiraceae*, *Roseburia* and *Ruminococcus*), were described [56].

The differences in bacterial diversity between countries may result from several factors, including genetic predisposition, diverse geographical locations and different nutrition and eating habits [57,58,59]. Our understanding of the profound impact of diet on microbiota composition, intestinal physiology and human health is rapidly increasing. Diet is a factor shaping the infant gut microbiome, as shown by the differences detected between breastfed and formula-fed babies in a twin cohort [58]. Multiple data indicate that a typical Western-style diet, high in animal protein, sugar, starch and fat and low in fiber, results in a specific intestinal microflora profile [57,58]. An interesting study compared gut microbiota sampled from children living in poor, rural areas (Burkina Faso; BF), whose food is low in fat and animal protein and rich in starch, fiber and plant polysaccharides, with that of European children (Florence). Children fed a rural African diet had a higher abundance of Actinobacteria and Bacteroidetes, whereas Firmicutes and Proteobacteria were more abundant in European children [57]. In consequence, Gram-negative bacteria dominated over Gram-positive bacteria in the BF population, whereas Gram-positive species were three times more abundant than Gram-negative among European children [57]. Furthermore, short-chain fatty acid (SCFA)-producing bacteria dominated in specimens from BF children. SCFAs are known to promote the integrity of the gut epithelial barrier and to regulate brain function, including histone deacetylase activity, microglial activation or the expression of growth factors [60]. It was suggested that a diet rich in plant polysaccharides and low in sugar and fat may promote the growth of bacterial species, protecting against gut inflammation [57]. 

Bacterial overgrowth in the small intestine occurs with high prevalence among PD patients, and its frequency ranges from 14 to even 67% [61]. In a meta-analysis, small intestine bacterial overgrowth correlated with PD and occurred with a higher prevalence in Western countries (52%) in comparison with Eastern countries (33%) [62]. Interestingly, intestinal microbiota composition in PD patients often shows resemblances to the changes detected in patients with inflammatory bowel disease [63]. 

Overall, the abundance of *Akkermansia*, *Enterococcus*, *Lactobacillus* and *Bifidobacterium* and a decrease in *Lachnospiraceae*, *Faecalibacterium* and *Roseburia* seem to be the most consistent findings in PD [51,64,65,66,67]. A reduction in the number of *Faecalibacterium* and *Roseburia* may lead to impaired production of SCFA [68]. The decrease in SCFA-producing bacteria and the increase in mucin-degrading *Akkermansia* may enhance intestinal permeability and, in turn, contribute to the transport of pro-inflammatory molecules into systemic circulation. In PD models, some SCFAs reduced the extent of neuronal degeneration and improved behavioral alterations [69]. 

The clear picture of bacterial dysbiosis in PD implies the potential therapeutic effect of antibiotic therapy. However, the available data are limited. Concomitant oral administration of ampicillin, neomycin and metronidazole evoked prominent changes in the composition of the microbiota and reduced MPTP-induced dopaminergic neurotoxicity in the brain [48]. Clinical trials in this area are basically absent. The ClinicalTrials.gov database revealed three studies (two completed and one still recruiting) assessing the effect of rifaximin in small groups of PD patients. Yet, to our knowledge, no results have been published so far. Furthermore, precision nutrition based on devising a diet beneficial in a given clinical scenario may become an important part of PD therapy.

In general, accumulated data imply that in PD, the pro-inflammatory gut environment, together with impaired mucosal barriers and “leaky gut” syndrome, may trigger an inflammatory response. Furthermore, increased α-synuclein synthesis and the subsequent transmission of misfolded α-synuclein to the brain follow [70]. Additionally, various enteric and microbial metabolites, hormones, immune molecules and activation of the vagus nerve affect the function of the brain and may induce or exacerbate the disease [31]. However, considering that each species carries specific metabolic characteristics, alterations in the composition of the microbiota may alter brain function in diverse ways. To ensure a more consistent pattern of changes, the combination of a well-defined food regimen and probiotic application seems required. We suggest that directing the microbiota metabolism towards Trp conversion may be of therapeutic value. 

## 3. Tryptophan Metabolism

**Figure 1:**
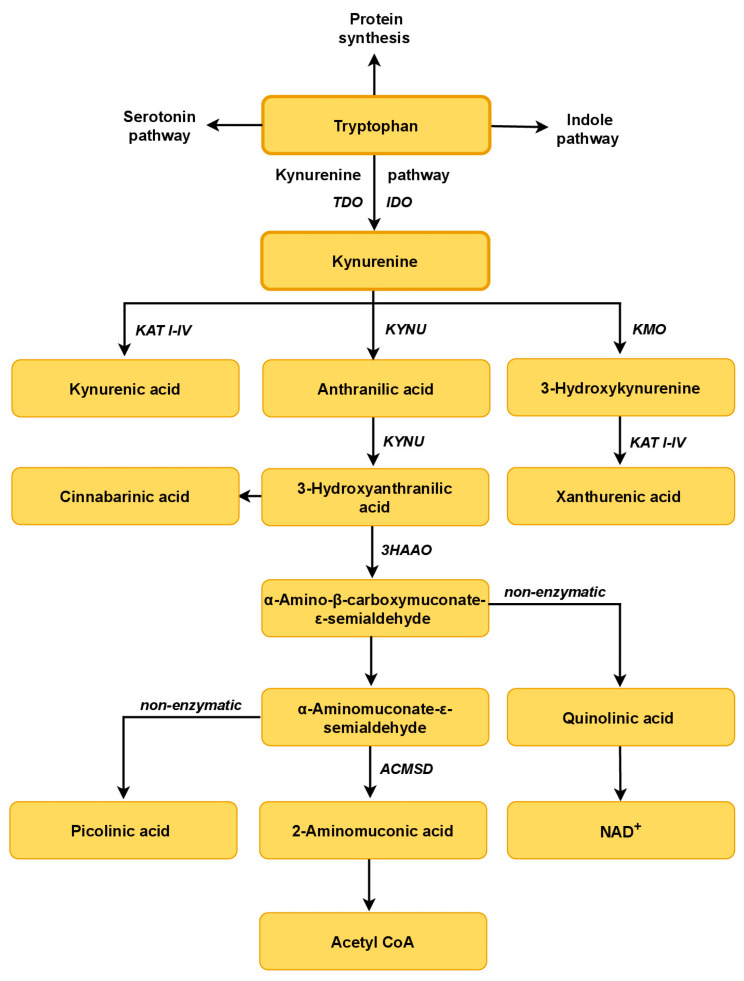
A schematic overview of tryptophan metabolic pathways. Kynurenine, serotonin and indole pathways are the primary routes of host Trp metabolism and its commensal bacteria. Kynurenine pathway: IDO, indoleamine 2,3-dioxygenase; TDO, tryptophan 2,3-dioxygenase; KAT I–IV, kynurenine aminotransferase I–IV; KMO, kynurenine 3-monooxygenase; KYNU, kynureninase; 3HAAO, 3-hydroxyanthranilate 3,4-dioxygenase; ACMSD, α-amino-β-carboxymuconate-ε-semialdehyde decarboxylase.

Trp enters a complex metabolic pathway, yielding compounds that contribute to several physiological processes, from neuronal function through immunity to metabolic homeostasis [71,72]. Trp availability plays a rate-limiting role in protein synthesis [73], yet only 1–2% of digested Trp is used for this purpose. The major route of Trp metabolism in the periphery and the brain comprises the Trp-KYN pathway, which converts approximately 95% of the Trp pool [21]. The remaining 2–3% of Trp enters the serotonin or indole pathways (Figure 1). Trp may pass the blood–brain barrier (BBB) only as a free, unbound form, constituting approx. 10–15% of its serum pool. Transport of Trp across the BBB is facilitated by the non-specific large neutral amino acid transporter (LNAAT) [74].

### 3.1. The Tryptophan–Kynurenine Pathway

The Trp-KYN pathway is functional in the microbiota and the human host. It starts with the conversion of Trp by Trp 2,3-dioxygenase (TDO) or indoleamine 2,3-dioxygenases (IDO1 and IDO2), synthesizing N-formylkynurenine [21,75,76]. IDO1 is expressed in the microglia, neurons and astrocytes, as well as in macrophages, fibroblasts and epithelial cells [77]. TDO, a rate-limiting enzyme, is detected mainly in hepatocytes and can be upregulated by Trp itself or by glucocorticoids [77]. Higher availability of Trp and its increased supply to the liver lead to enhanced TDO activity and stimulation of the Trp-KYN pathway [71]. N-formylkynurenine is rapidly converted to L-KYN by formamidase. In the brain and periphery, L-KYN is metabolized along two major enzymatic branches: (A) generating kynurenic acid (KYNA) in the process catalyzed by four kynurenine aminotransferases (KATs I–IV), or (B) producing 3-hydroxykynurenine (3-HK) and anthranilic acid (AA) by kynurenine 3-monooxygenase (KMO) and kynureninase, respectively [78,79]. Kynureninase may also metabolize 3-HK to 3-hydroxyanthranilic acid (3-HANA). KAT II, and possibly other KATs, may synthesize xanthurenic acid (XA) from 3-HK [22,23]. 3-HANA may yield α-amino-α-carboxymuconic-ω-semialdehyde (ACMS) in the process catalyzed by 3-hydroxyanthranilic acid 3,4-dioxygenase (3HAO). Under physiological conditions, ACMS spontaneously rearranges to form quinolinic acid (QUIN). Condensation of two molecules of 3-HANA yields cinnabarinic acid [22,71]. QUIN serves as an intermediate in the production of NAD+, an essential enzymatic cofactor [22,71]. Among all of the molecules from the Trp-KYN pathway, only Trp and KYN may penetrate the blood–brain barrier [22,23].

Kynurenines display a wide range of divergent biological activities, including cytotoxic/cytoprotective, oxidative/antioxidant or pro-/anti-inflammatory. Classically, metabolites of the Trp-KYN pathway are divided into neuroprotective and neurotoxic groups. However, the action of various kynurenines may depend on the local availability of substrates, enzymatic activity and the broadly understood cellular environment [80,81]. Moreover, a complex network of endogenous factors as well as exogenous modifiers, e.g., the availability of Trp, presence of inflammation and exposure to environmental toxins may alter the ultimate role that a specific kynurenine plays. Thus, the original concept of protective or toxic kynurenines gradually evolves.

Metabolites generally accepted as neuroprotective include KYN, KYNA, XA and picolinic acid. The beneficial effects of KYN observed in various experimental models have traditionally been attributed to KYNA production. However, KYN itself has redox properties as a scavenger of free radicals such as peroxynitrite (ONOO−), superoxide anion (O2^•−^) and hydroxyl radicals (OH radicals) [23]. KYN has been identified as an endogenous aryl hydrocarbon receptor (AhR) ligand that functions as a xenobiotic target and transcription factor [78]. 

KYNA, a direct transamination product of KYN, is an important metabolite of the Trp-KYN pathway that exhibits pleiotropic biological activity [76]. KYNA is a well-known neuroprotective, anti-epileptic and anti-inflammatory compound that blocks ionotropic glutamate receptors. It displays the highest affinity toward the glycine site of the N-methyl-D-aspartate (NMDA) receptors. KYNA was also identified as an agonist of AhR and G protein-coupled orphan receptor 35 (GPR35) [76]. The deficiency of KYNA was implicated as an important factor in the development of various metabolic, cardiovascular or brain disorders [21,23].

XA has antioxidant properties and is a free radical scavenger. Picolinic acid acts as an effective chelator of metals (such as chromium, zinc, manganese, copper and iron) preventing protein aggregation and oxidative stress [82]. 

Metabolites of KP with prevailing neurotoxic properties include 3-HK, QUIN and 3-HANA. 3-HK is capable of inducing oxidative damage and cell death and has thus been implicated in various neurological and psychiatric disorders [83]. Increased levels of 3-HK in the brain have been associated with several neurodegenerative diseases, including Huntington’s disease [84]. However, some experimental studies have provided evidence of the antioxidant properties of this molecule. At low levels, 3-HK acts as an antioxidant and can scavenge reactive oxygen species (ROS) such as superoxide radicals [85]. 

Similarly, 3-HAA can induce oxidative stress and promote ROS synthesis [86]. On the other hand, in primary glial cultures exposed to cytokines, 3-HAA induced the expression of hemeoxygenase-1, which has anti-inflammatory and cytoprotective properties [87].

QUIN is a classical excitotoxin, acting at the agonist-binding site of the NMDA receptor at higher concentrations, whereas at low, physiological levels, it serves as an intermediate for the production of NAD+. However, during long-lasting exposure to QUIN, the compound may induce neuronal loss even at low concentrations [76]. Moreover, QUIN may stimulate glutamate release and inhibit its reuptake, thus potentiating glutamate-mediated neurotoxicity [76]. Like many other kynurenines, QUIN was also reported to promote lipid peroxidation [76].

Some Trp metabolites were reported to modify the physical and chemical properties of proteins, which results in their altered structure and function, such as unfolding, cross-linking or changing solubility [88]. 3-HK-modified proteins were found in the urine of hemodialyzed patients, manifesting elevated levels of kynurenines in the periphery [89,90]. Furthermore, it was demonstrated that KYN-derived chromophores interact with α-1-microglobulin in the lens and urine, which may be involved in the development of cataracts and exacerbate uremia [90,91]. Additionally, cross-linked KYN-modified proteins are photosensitizers and can produce free oxygen radicals [92,93].

### 3.2. Trp Metabolism in the Microbiota–Indole Pathway

Various gut species, including *Lactobacillus*, *Bifidobacterium*, *Bacteroides* and *Clostridium*, can metabolize Trp [25]. Microbiota may convert Trp along the Trp-KYN pathway, mainly within immune and epithelial cells [94], along the serotonin pathway in enterochromaffin cells [95] and into several other molecules, including ligands of the AhR. The Trp-KYN and serotonin pathways in microbiota are analogous to those in human hosts. However, in contrast to the human host, intestinal microorganisms can metabolize Trp via the indole biosynthetic pathway [96,97]. 

Tryptophanase, expressed in various bacteria, including *Escherichia coli*, *Clostridium* spp. and *Bacteroides* spp., hydrolyzes Trp to indole [98]. Indole can be released and subsequently absorbed by the host cells. In the liver, xenobiotic metabolizing enzymes, cytochrome P450 and sulfotransferases convert indole into compounds such as indoxyl-3-sulfate (IS), oxindole and isatin [99]. Trp can also be degraded by intestinal bacteria to indole-3-propionic acid (IPA), indole-3-acetaldehyde (IAld), indole-3-acetic acid (IAA), indole-3-lactic acid (ILA), tryptamine, indoleethanol (IE), indole-3-acetaldehyde (IAAld) or 3-methylindole (skatole) [100]. There are qualitative and quantitative differences in indole production among different people due to diverse gut microbiota species colonizing the gut.

The oxidative and reductive pathways in *Clostridium sporogenes* lead to the production of IAA and IPA [101,102]. *Clostridium bartlettii* and *Bifidobacterium* spp. produce ILA and IAA [103]. *Peptostreptococcus* spp. converts Trp to IA and IPA. IAld is also generated by *Firmicutes* species such as *Lactobacillus (L.) reuteri*, *L. johnsonii*, *L. acidophilus* and *L. murinus* [103] via the aromatic amino acid aminotransferase (ArAT) and an indolelactic acid dehydrogenase (ILDH)]. Skatoles are generated by the decarboxylation of IAA by *Bacteroides* spp. and *Clostridium* spp. [103]. *Ruminococcus gnavus* converts Trp into tryptamine using Trp decarboxylase. 

Indole, IPA and IAld promote the maintenance of the epithelial barrier, enhance the secretion of mucus, increase the thickness of the mucus layer, improve gut barrier function and alleviate inflammatory reactions. Pregnane X receptor (PXR), Toll-like receptor 4 (TLR-4) and AhR seem to be involved in the reduced intestinal permeability and decreased inflammation [104]. Thus, increasing the synthesis of AhR ligands along the Trp-KYN pathway should result in the diminution of the “leaky gut” syndrome and alleviate local immune alterations. In turn, decreased absorption of potentially harmful compounds, derived from nutritional sources as well as from microbiota metabolism, should exert a positive impact on brain function. 

### 3.3. Trp-KYN Pathway in PD

The shift of the Trp-KYN pathway to either neurotoxic kynurenines such as 3-HK or QUIN or to the neuroprotective KYNA profoundly affects neuronal function and survival [76,105]. Under physiological conditions, the astrocytic Trp-KYN pathway serves as a source of neuroprotective KYNA, while the neuronal pathway produces NAD+, thereby improving the energy status of the cell [106,107]. Inflammatory responses to cytotoxic events triggered by various mechanisms start a cascade of events that, in the end, augment the initial insult. One of the underlying mechanisms is linked with the induction of IDO and subsequent stimulation of the Trp-KYN pathway, leading to an overabundance of neurotoxic kynurenines. In the brain, the net effect of these processes can be viewed as the interplay between astrocytes, microglia and neurons.

In 1983, Prof. Langston and his group discovered that administration of MPTP, a highly lipophilic toxin that readily reaches the brain, induces SN degeneration followed by a PD-like behavioral pattern (see, for review, [108]). Selective toxicity depends primarily on the glial conversion of MPTP to a pyridinium metabolite (1-methyl-4-phenylpyridinium; MPP^+^), which inhibits the neuronal mitochondrial respiratory chain and is a source of free radicals [85]. MPP^+^ inhibits cortical KAT activity and reduces KYNA formation in vitro and in vivo [109,110]. KYNA was shown to prevent neurodegeneration in an MPTP model of PD disease. It also downregulates Bax expression and protects human neuroblastoma cells against MPP^+^-induced degeneration [98,111,112]. Similarly, the application of probenecid and KYN, causing an increase in brain KYNA, was shown to reduce 6-OHDA toxicity, neuronal damage and behavioral changes [113]. Furthermore, the administration of a KMO inhibitor, resulting in higher availability of KYN and increased KYNA formation, alleviated L-DOPA-induced dyskinesias [114]. Thus, modifications of Trp metabolism appear to have a dual therapeutic benefit in PD: neuroprotection and prevention of L-DOPA-induced motor side effects [115]. Overall, experimental animal data suggest that in the brain, the shift of the Trp-KYN pathway to the branch producing 3-HK and QUIN, accompanied by a relative or complete KYNA deficiency, is an important factor contributing to the development and progression of PD. 

Furthermore, experimental data revealed that in 6-OHDA and MPTP models of PD, the deficit in KYNA formation may result from the diminished expression of neuronal KAT I in neurons of SN pars compacta [110,116]. Considering that chronic MPTP administration increases the expression of IDO in the striatum [117], activation of the kynurenine pathway in neurons seems to result in a deficiency of neuroprotective and an overabundance of neurotoxic metabolites. Indeed, accumulated data support the concept that enhanced activity of IDO and TDO contributes to motor and behavioral disturbances as well as gastrointestinal symptoms in PD [105,118]. In contrast, in the SN of rotenone-treated rats, a reduced expression of kynureninase, generating 3-HK, was reported [119]. Further research should clarify the precise central changes in the expression of KP enzymes in the models of disease.

Despite the quite clear picture arising from animal data, the results of clinical research are equivocal. In the periphery, both decreased and increased levels of kynurenines were reported. Higher KYNA and KAT II activity were detected in PD patients’ erythrocytes, together with an increased KYN/Trp ratio, lower plasma Trp and higher KYN [120]. Others did not reveal changes in serum KYNA levels; however, decreased activities of KAT I and KAT II were found [121]. A lower plasma KYNA/L-KYN ratio, higher QUIN levels, an increased QUIN/KYNA ratio and high 3-HK were reported [122,123]. In contrast, no changes in the levels of KYN, KYNA, AA and 3-HK in either the serum or CSF of PD were shown [124]. On the other hand, decreased KYNA and increased QUIN were reported in the CSF of PD patients [96]. A meta-analysis including ten studies, involving 539 participants (265 patients and 274 controls), revealed that in PD, the level of Trp in the blood, but not in the cerebrospinal fluid (CSF), is significantly lower compared to the control group. In the brains of PD patients, KYN and KYNA levels are decreased in the frontal cortex, putamen and SN [125]. A recent study revealed that SNPs in the rate-limiting enzyme of the Trp-KYN pathway, IDO1, may impact the onset of PD [105].

As mentioned above, α-synuclein, misfolded and accumulated in Levy bodies, is implicated in the pathogenesis of synucleinopathies and is considered a hallmark of PD [126]. Aberrant soluble oligomers may disrupt cellular homeostasis and induce neuronal death through various intracellular targets. Furthermore, secreted α-synuclein can damage neighboring cells through the seeding of aggregates and subsequently further propagate PD [127]. So far, the triggers evoking aggregation of α-synuclein are not well recognized. The intriguing possibility that kynurenines contribute to this process is supported by the data from experiments in SH-SY5Y cell cultures. QUIN stimulated the generation of amyloid-like assemblies and the seeding of α-synuclein aggregates in vitro [128]. Furthermore, it was shown that 3-HK and its auto-oxidation products may generate derivatives of α-synuclein in a process boosted by the oxidizing environment [129]. 

The above data indicate that the Trp-KYN pathway plays an important role in the development of PD. The areas of the brain profoundly affected by neurodegeneration produce smaller quantities of KYN and KYNA. In the periphery, however, the status of the Trp-KYN pathway varies and may depend on the stage of PD, comorbidities, accompanying inflammation and environmental factors. It is conceivable that the peripheral conversion of Trp to kynurenines is in part regulated by the microbiota. The levels of kynurenines in the blood are linked not only with the metabolism of Trp in peripheral organs but also reflect the activity of the microbiota in converting Trp. Subsequent absorption of metabolites through the intestinal wall may prominently affect concentrations of kynurenines in systemic circulation. 

In the gut, free Trp from dietary proteins is absorbed within the small intestine into the systemic circulation. The remaining Trp is transported into the colon and utilized by the microbiota. Indeed, germ-free mice, completely deprived of all commensal microorganisms, exhibit high central and peripheral Trp levels [130]. Moreover, as mentioned before, antibiotic therapy leading to the eradication of microbiota was shown to alleviate MPTP-induced neurotoxicity [48]. The beneficial effects of microbiota depletion may result from the modulation of the availability of serum Trp and its metabolites.

## 4. AhR in the Gut–Brain Axis

AhR, a ligand-activated transcription factor, is an 848-amino-acid-long protein encoded by a gene located on chromosomes 7 and 12 in mice and humans, respectively [131]. Although AhR was initially recognized as a mediator of the toxicity of xenobiotics, its essential role in the regulation of various biological processes, including immune response, is now well acknowledged [132,133]. Dietary, microbial or metabolic signals may impact AhR signaling and thus modulate diverse biological processes [131].

**Figure 2:**
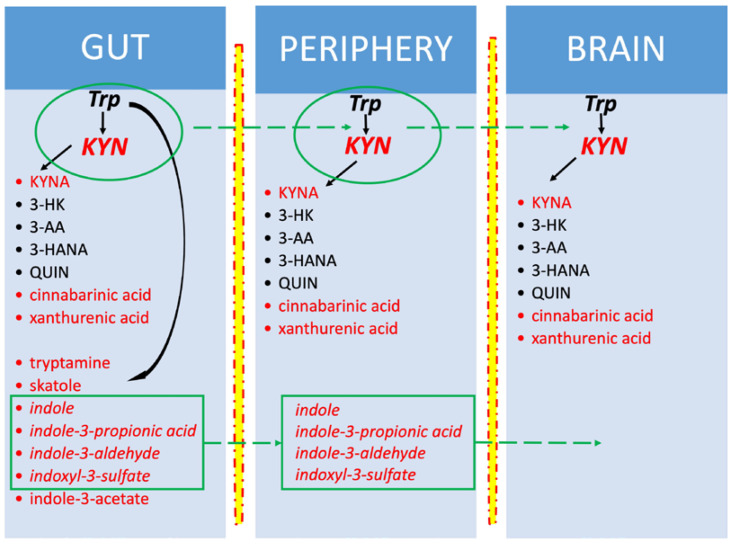
Tryptophan metabolites generated in the gut, periphery and brain. In red—AhR ligands; in italics—metabolites penetrating through the blood–brain barrier. Tryptophan (Trp) and kynurenine (KYN) are also easily transported into the brain. Kynurenines are produced in all three compartments, in contrast to indoles, which are synthesized only in the gut. In the brain, Trp comes from ingested proteins, and KYN originates from Trp metabolism in the gut and peripheral organs and from the conversion of Trp in situ. Other brain kynurenines are produced locally within the brain.

Trp is an important source of AhR ligands. As mentioned above, degradation of Trp by bacteria or enterocytes generates several AhR-binding molecules such as KYN, KYNA, cinnabarinic acid, xanthurenic acid, tryptamine and indoles: 2-(19H-indole-39-carbonyl)-thiazole-4-carboxylic acid methyl ester (ITE), 3-methylindole (skatole), 6-formylindolo [3,2-b] carbazole, indole-3-aldehyde (IAld), indoxyl-3-sulfate (I3S) [131,132]. Indole, indoxyl-3-sulfate, indole-3-propionic acid and indole-3-aldehyde can cross the blood–brain barrier [133] (Figure 2).

### 4.1. AhR Signaling Pathways

AhR binds multiple compounds, including dietary, commensal and endogenous ligands. Apart from xenobiotics such as 2,3,7,8-tetrachlorodibenzo-p-dioxin (TCDD), dibenzofurans, biphenyls, benzopyrene or benzanthracenes, food-derived compounds, metabolites of commensal microbiota and certain drugs (e.g., sulindac or diclofenac) are also able to act as AhR ligands. It is of interest that dopamine itself and carbidopa, used concomitantly with levodopa in the therapy of PD, are ligands of AhR [134].

The inactive form of AhR is located in the cytoplasm and bound with actin filaments, chaperone proteins including heat shock protein 90 (HSP90), protein kinase c-Src, AhR-interacting protein (AIP) and co-chaperone p23. Two signaling pathways mediate the AhR response. The genomic path is triggered by agonist binding, followed by the release of the AhR complex from actin filaments and its translocation into the nucleus. Further, AhR interacts with DNA sequences, i.e., xenobiotic responsive elements (XREs, also known as DREs), in the regulatory regions of target genes (e.g., *CYP1A1*, *CYP1A2*, *CYP1B1*). Activation of CYP results in the degradation of AHR ligands and constitutes a negative feedback loop that restricts AhR activation. AhR may also interact with other transcription factors such as the pro-inflammatory nuclear factor kappa-light-chain enhancer of activated B cells (NF-κB), estrogen receptor or retinoic acid receptor. NF-κB plays a crucial role in the regulation of inflammation, apoptosis and aging and has been implicated in the pathogenesis of PD [135,136]. The ability of AhR to exert epigenetic effects was also shown [131]. Non-genomic ways include, among others, an interaction with the Trp-KYN pathway. Released from the complex with AhR, the protein kinase c-Src may phosphorylate and stabilize IDO1, thus affecting the fate of Trp metabolism [137].

### 4.2. AhR in PD

In the CNS of rodents, transcriptionally active AhR and its mRNA were detected in all brain regions within astrocytes, microglia and neurons [138,139,140]. However, the precise role of AhR in the brain still awaits elucidation. One of the major obstacles originates from the dual activity of AhR. Depending on the ligand, AhR may mediate either pro-inflammatory (through NF-κB) or anti-inflammatory effects [140]. 

AhR agonists derived from the microbial metabolism of dietary Trp were demonstrated to attenuate the pro-inflammatory phenotype of glia [141]. In mice, Trp metabolites, indole, indoxyl-3-sulfate, indole-3-propionic acid and indole-3-aldehyde reduced inflammation and experimental autoimmune encephalomyelitis disease scores [139]. Type I interferons produced in the brain seem to act in concert with Trp metabolites produced by the gut microbiota to activate AhR signaling in astrocytes and suppress CNS inflammation [139].

The data on the role of AhR in PD are, to our knowledge, limited to experimental animal research. In the MPTP-induced model, the number of AhR-positive microglial and glial cells in the striatum and the SNpc increased, whereas AhR-positive tyrosine-hydroxylase-expressing neurons were reduced [100]. In mice, the administration of the AhR ligand resulted in a twofold increase in protein levels of a ubiquitin-conjugating enzyme that interacts with parkin (UbcH7). The AhR ligand stimulated UbcH7 expression only in dopaminergic neurons and decreased levels of synphilin-1 protein in the ventral midbrain. Synphilin-1 interacts with α-synuclein and is linked with familial PD and SN neurodegeneration [138,142,143]. In line with the above observations, the administration of another AhR ligand, diindolylmethane, prevented the loss of nigral dopaminergic neurons in the MPTP model [144]. Thus, in the above paradigms, AhR stimulation seems to fuel the anti-inflammatory state and exert beneficial effects in PD.

So far, three synthetic AhR agonists—laquinimod, tranilast and benvitimod—have been investigated in phase I–III clinical trials. The trials involved patients with autoimmune conditions, such as Crohn’s disease, rheumatoid arthritis, asthma, atopic dermatitis or multiple sclerosis [145]. However, to our knowledge, none of them was tested in PD patients.

It is conceivable that, depending on the composition of the intestinal microbiota, the proportion of various gut-derived kynurenines will vary. Considering the alterations in the microbiota detected in PD, we may hypothesize that the production and absorption of AhR ligands, especially KYN and KYNA, into the systemic circulation are impaired in these patients. This, in turn, would affect the delivery of metabolites easily penetrating the BBB, such as KYN, to the brain and affect neuronal survival among individuals at risk of PD.

We suggest that the peripheral levels of kynurenines are strongly linked to the Trp metabolism in the gut and, as such, should be studied together with the composition of the microbiota. Only such an approach can clearly delineate the sub-populations of PD patients manifesting with a disturbed microbiota/Trp-KYN/brain triad who would benefit from modifications of Trp metabolism.

## 5. Dietary Interventions

The recommended dietary allowance of Trp is estimated to be in the range of 3.5–6 mg/kg, i.e., 250–500 mg/day [73,146]. Products richest in Trp include various seeds, nuts and cocoa. Trp content per 100 g of products ranges from 590 mg/100 g (pumpkin seeds), 370 mg/100 g (sesame seeds) and 240 mg/100 g (cashew nuts) to 290 mg/100 g (cocoa). Animal products with high Trp content include milk (550–800 mg/l) and poultry meat (100–150 mg/100 g) [73,146]. For a detailed review of the content of Trp and other kynurenines in food products, see [147].

Studies on the potential beneficial effects of Trp were performed in a broad range of experimental paradigms [146,148]. However, the results are not conclusive. Various modalities, such as age, diet, comorbidities, currently taken supplements and drugs, may all modify the outcome of such approaches. Research concerning the impact of Trp intake on its peripheral and central metabolism indicates the potential value of dietary interventions in neurological conditions. 

### 5.1. Oral Tryptophan and Peripheral Kynurenines

Low-, medium- and high-Trp diets (0.6, 1.2 or 1.8%) fed to rats for 3 months resulted in changes in the levels of AhR and kynurenines [149]. In the high-Trp group, serum levels of AhR, KYN, 5-hydroxyindole-3-acetic acid, indole-3-lactic acid, indoleacetate and indoxyl sulfate were increased [149]. A randomized clinical study in 17 healthy women receiving from 1 to 5 g of Trp showed that the urinary excretion of Trp and other metabolites, such as KYN, KYNA, 3-HK and 3-HANA, increases dose-dependently during dietary intervention [150]. A study in 40 elderly persons with insomnia or depression revealed that patients’ initial dietary Trp intake is low, their urinary excretion of KYNA is unaltered and excretion of KYN and QUIN is high. A prolonged, 12-week intake of 25 mg/kg of Trp daily resulted in mood improvement and increased elimination of Trp but reduced the elimination of KYN and QUIN with urine [151]. A double-blind, placebo-controlled study showed prominent increases in plasma KYN, KYNA and 5-HIAA, occurring 4 h after the oral administration of a single dose of Trp (6 g) to patients with schizophrenia as well as in healthy individuals [152,153]. 

### 5.2. Oral Tryptophan and Brain Kynurenines

The impact of Trp supplementation on the Trp-KYN pathway in the brain is complex. A properly balanced diet with an appropriate intake of proteins seems at first like an optimal approach to maintaining an adequate Trp supply. However, in contrast to the intuitive expectation, providing a high-protein diet does not result in an increased brain Trp level. A higher amount of Trp absorbed from the gut stimulates the activity of liver TDO and hepatic conversion of Trp, thus reducing its availability. This, together with increased levels of other protein-derived LNAA, which compete for the LNAAT activity in the BBB, limits the amount of Trp entering the brain [148]. On the contrary, a high-carbohydrate diet improves brain Trp availability through stimulation of insulin release. Insulin enhances the uptake of branched-chain amino acids into muscles, thus improving the Trp/LNAA ratio [148]. Furthermore, a dietary supply of high levels of polyunsaturated fatty acids (PUFAs) may increase the free fraction of Trp due to its displacement from albumin-binding sites. Interestingly, PUFA supplementation correlates inversely with plasma 3-HK levels and the KYN/Trp ratio [154].

Much less is known about brain kynurenines following peripheral Trp administration. In rats receiving a high Trp content diet for 24 h (1.5% Trp), a 3-fold increase in extracellular KYNA levels (320% of control) was demonstrated [155]. To our knowledge, there are no available data concerning CSF or brain levels of kynurenines following controlled oral supplementation with Trp in humans. 

Interestingly, physical exercise in the elderly raised the free serum Trp levels [156]. Having the above in mind, it may be assumed that exercise would be a safe way to improve the uptake of Trp into the brain. Exercise, through increased lipolysis, yields high levels of non-esterified fatty acids, which compete with serum albumins and displace Trp from the albumin-binding sites. This will increase the brain availability of Trp [148]. Future studies should assess this intriguing probability that enhanced uptake of Trp into the brain may be one of the beneficial mechanisms of exercise for neuronal survival. 

Moreover, accumulating data emphasize that exercise has a profound impact on the intestinal microbiome [157,158,159]. Improved colon function, increased microbiota diversity and the restored balance between beneficial and pathogenic bacterial communities were reported [160,161]. A recent systematic review identified Firmicutes and Actinobacteria as the main exercise-responsive phyla [162]. Furthermore, exercise may increase the number of SCFA-producing bacteria in both rodents and humans [163,164]. Moreover, preclinical studies have shown that exercise increases the activity of key antioxidant enzymes (catalase and glutathione peroxidase), anti-inflammatory cytokines (including interleukin 10; IL-10) and anti-apoptotic proteins (including Bcl-2) in intestinal lymphocytes, while reducing the level of pro-inflammatory cytokines (TNF-α and IL-17) and pro-apoptotic proteins (caspases 3 and 7) [165,166]. As a result, exercise may lead to an overall reduction in inflammation and the subsequent improvement of existing PD. It seems conceivable that maintaining adequate physical activity, regardless of age, may delay or even prevent the development of PD.

### 5.3. Antibiotics and Probiotics

In line with the concept of the beneficial effects of Trp, a low-Trp diet was demonstrated to produce systemic inflammation in experimental animals. Moreover, animals fed a low-Trp diet (0.1%) showed altered bacterial composition in the gut microbiota compared to animals receiving a control (0.2%) or high-Trp (1.25%) diet [167]. The abundance of *Acetatifactor*, *Enterorhabdus* and *Adlercreutzia* was increased and Deferribacteres were decreased in a low-Trp group [167]. In the Trp-deficient diet, serum IL-6 and IL-1a were upregulated, whereas anti-inflammatory IL-27 was decreased [167]. Notably, *Acetatifactor muris* transplanted into control mice may cause colon inflammation [168].

Considering that microbiota efficiently break down Trp, it is conceivable that targeting the gut microbiota may profoundly alter the availability of Trp. The resulting changes in Trp metabolism will depend on a combination of factors: (a) microbiota species that are prevalent in the gut; (b) the presence or lack of inflammation in the intestinal wall; (c) the composition of food, and other aspects, such as age, physical activity or genetic determinants.

Disturbed proportions between intestinal microbiota species follow antibiotic therapy and result in changes in gut metabolism. Increased serum Trp and decreased KYN, a higher Trp/KYN ratio and a higher KYNA/KYN ratio were observed in the experimental administration of antibiotics in mice, probably as a result of the increased availability of Trp for peripheral metabolism [169]. A mixture of ampicillin, vancomycin, neomycin and metronidazole, supplemented with amphotericin-B and administered from the 21st postnatal day, reduced the fecal bacterial DNA load by 400-fold. Furthermore, a decreased abundance of Firmicutes and Bacteroidetes and an increased quantity of Cyanobacteria and Proteobacteria were reported [169]. Similarly, a decline in serum Trp was reported in other studies evaluating orally administered antibiotics in pigs [170,171,172,173].

On the other hand, probiotics from the genera *Lactobacillus* and *Bifidobacterium* may inhibit the metabolism of Trp towards kynurenines with a concomitant increase in indole metabolites [25,174]. A decrease in serum KYN and lower IDO activity were described in rats and humans receiving *Lactobacillus* or *Bifidobacteria* [25]. *Lactobacillus reuteri* also produce high amounts of AhR ligands—IAld, ILA and IAA [175]. In humans, a significant decrease in plasma KYN concentration, an increased 3-HK/KYN ratio and no changes in Trp or KYNA levels were reported after administration of *Lactobacillus plantarum 299v* for 8 weeks [176].

Thus, certain probiotics seem to reduce the conversion of Trp to kynurenines, whereas the synthesis of microbiota-derived AhR ligands increases. Boosted production of AhR ligands appears to be an important factor that connects the microbiota with the Trp-KYN pathway and brain function. It becomes clear that the modulation of microbiota composition may be an important tool allowing for controlling Trp availability for the host. Intervention with probiotics seems like an attractive way to modulate Trp metabolism, yet it requires further, detailed studies concerning the choice of optimal bacterial strain(s) able to exert therapeutic effects.

Apart from direct modulation of the intestinal microbiota by means of probiotic application, a low-protein, high-carbohydrate (LPHC) diet seems like a promising dietary intervention in neurodegeneration. In MPTP-induced parkinsonism in mice, the LPHC diet normalized the microbiota composition imbalance by increasing the abundance of *Bifidobacterium*, *Ileibacterium*, *Turicibacter* and *Blautia* and decreasing *Bilophila*, *Alistipes* and *Bacteroides*. Apart from ameliorating MPTP-induced motor deficits and reducing neuronal loss, the LPHC diet exerted several metabolic effects, including a rise in serum Trp [177]. 

### 5.4. Safety of Dietary Supplementation with Tryptophan

Administration of Trp in the form of supplementation should not exceed 4.5 g/d, as proposed for young adults [178]. Coexisting disorders may constitute relative or absolute contraindications for increased Trp supply with the diet. The major concern should be the proper function of the kidney, which should be assessed before supplementation. In an experimental model of chronic kidney injury, administration of Trp (500 mg/kg for 8 weeks) increased mortality and serum creatinine and enhanced renal damage in rats [149]. It is suggested that the accumulation of Trp-derived uremic toxins in humans, caused by excessive Trp intake, may influence the progression of chronic kidney disease [179]. 

### 5.5. Fecal Microbiota Transplantation

Manipulating gut microbiota through procedures such as fecal microbiota transplantation (FMT) has received increasing attention [180]. Beneficial effects of FMT include preserved intestinal barrier function, improved intestinal motility, production of SCFA or a reduced number of pro-inflammatory bacterial strains in the gut [181,182,183]. The potential benefit of FMT was reported, e.g., in obesity, cardiovascular disorders, epilepsy and cognitive impairment [184,185,186,187]. In the animal MPTP model of PD, FMT was shown to improve the composition of the gut microbiota, decrease α-synuclein expression, inhibit microglial activation in SN, block pro-inflammatory signaling pathways and reduce behavioral changes [188,189]. Clinical data on FMT in PD are still limited. A preliminary study performed in a group of 15 PD patients revealed that FMT via colonoscopy (N = 10), in contrast to nasal–jejunal transplant (N = 5), alleviated the symptoms of the disease 1 month after the procedure [190]. Another small prospective study in 11 PD patients showed improvement in motor and non-motor functions after FMT was delivered through a nasoduodenal tube [191]. In light of the above data, future research seems vital in order to evaluate the potential long-lasting benefits and precise dosing regimens of FMT in PD.

## 6. Future Perspectives and Conclusions

**Figure 3:**
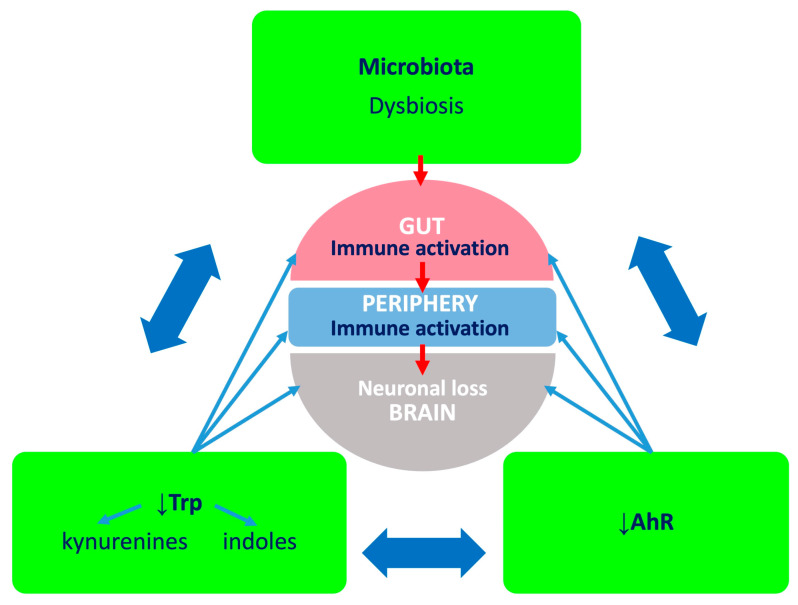
Microbiota, tryptophan (Trp), aryl hydrocarbon receptor (AhR) triad and the microbiota–gut–brain axis. Dysbiosis may affect Trp metabolism in the gut and the production of AhR ligands. Ensuing pro-inflammatory environment impacts intestinal enterocytes, and cytokines and chemokines evoke inflammation in situ. Furthermore, when absorbed into the systemic circulation, inflammatory molecules affect peripheral organs and the brain. Deficiency of Trp and AhR ligands further exacerbates inflammation. This, together with the disturbed metabolic conversion of Trp and shift from neuroprotective to neurotoxic kynurenines, will contribute to cytotoxicity.

Accumulated evidence supports the concept that microbiota, Trp and AhR form a target triad in PD (Figure 3). The current treatment of PD is primarily symptomatic and aimed at boosting impaired central dopaminergic neurotransmission, yet we still do not possess therapeutic options able to impact the cellular degeneration. Considering that the abnormal composition of intestinal flora is a prominent feature of the PD phenotype, oral supplementation with Trp together with probiotics and drugs modifying the Trp-KYN metabolism pathway may become a valuable, novel therapeutic approach. However, prominent individual differences, as well as the impact of comorbidities, food patterns, medications, infections or even lifestyle can modify the composition of the gut microbiota. Therefore, detailed research using a rigorous experimental approach, considering the above, should be carried out in the future.

Firstly, there is a need for detailed studies evaluating the impact of probiotics on Trp metabolism towards cytoprotective kynurenines and AhR ligands. The research should focus on the short- and long-term oral application of different microbiota species, with and without Trp supplementation, and studies of the intestinal, peripheral and central metabolisms of Trp. The influence of food regimens on the outcome should also be assessed. Considering the profound inter- and intra-species differences in intestinal colonization, research should be conducted initially under strictly regulated food patterns. In the second stage, the experimental approach should be aimed at comparing whether, despite different genetic backgrounds, genders and food compositions, the probiotic–Trp combo will still be effective.

Secondly, the effect of Trp–probiotic supplementation should be explored clinically in larger cohorts of individuals as an intervention aimed at changing the peripheral and central Trp metabolism. Even though precisely defined and customized diets appear promising regarding their impact on microbiome and inflammation, their everyday use in the general population is questionable. Thus, the research should focus on selecting the optimal proportion, frequency and duration of Trp–probiotic administration that would be effective in various paradigms.

Thirdly, the analyses of intestinal microbiota composition and intestinal and serum levels of kynurenines and indoles should be assessed as possible screening and diagnostic tests in a broader population, together with parallel neuroimaging analyses and cognitive assessments. We expect that such an approach may allow early detection of individuals at higher risk of developing PD, enabling personalized treatment. Therapies targeting enzymes or products of the kynurenine pathway may halt or slow dopaminergic degeneration.

Finally, the research on microbiota, Trp-KYN pathway metabolites and AhR signaling may shed light on the mechanisms of intestinal distress and identify new targets for the diagnosis and treatment of this condition in early-stage PD.

## Data Availability

Not applicable.

## Abbreviations

3HAAO	3-hydroxyanthranilate 3,4-dioxygenase
3-HANA	3-hydroxyanthranilic acid
3-HAO	3-hydroxyanthranilic acid 3,4-dioxygenase
3-HK	3-hydroxykynurenine
6-OHDA	6-hydroxydopamine
AA	anthranilic acid
ACMS	α-amino-α-carboxymuconate-ε-semialdehyde
ACMSD	α-amino-β-carboxymuconate-ε-semialdehyde decarboxylase
AhR	aryl hydrocarbon receptor
AIP	protein kinase c-Scr AhR-interacting protein
ArAT	aromatic amino acid aminotransferase
BBB	Blood–brain barrier
CNS	central nervous system
CSF	cerebrospinal fluid
CYP	cytochrome P450
FMT	fecal microbiota transplantation
GPR35	G-protein-coupled orphan receptor 35
HSP90	heat shock protein 90
I3S	indoxyl-3-sulfate
IAA	indole-3-acetic acid
IAAld	indole-3-acetaldehyde
IDO	indoleamine 2,3-dioxygenase
IE	indole ethanol
IL	interleukin
ILA	indole-3-lactic acid
ILDH	indole lactic acid dehydrogenase
IPA	indole-3-propionic acid
IS	indoxyl-3-sulfate
ITE	2-(19H-indole-39-carbonyl)-thiazole-4-carboxylic acid methyl ester
KAT I–IV	kynurenine aminotransferase I–IV
KMO	kynurenine 3-monooxygenase
KYN	kynurenine
KYNA	kynurenic acid
KYNU	kynureninase
L-DOPA	levodopa
LNAA	large neutral amino acid
LNAAT	large neutral amino acid transporter
LPHC	low-protein, high-carbohydrate
LPS	lipopolysaccharide
MHC	major histocompatibility complex
MIND	diet Mediterranean-DASH Intervention for Neurodegenerative Delay
MPP+	1-methyl-4-phenylpyridinium ion
MPTP	1-methyl-4-phenyl-1,2,3,6-tetrahydropyridine
NAD+	nicotinamide adenine dinucleotide
NF-κB	nuclear factor kappa-light-chain enhancer of activated B cells
NMDA	N-methyl-D-aspartate
PD	Parkinson’s disease
PUFAs	polyunsaturated fatty acids
PXR	pregnane X receptor
QUIN	quinolinic acid
ROS	reactive oxygen species
SCFA	short-chain fatty acid
SN	substantia nigra
Src	Src family kinases
TCDD	2,3,7,8-tetrachlorodibenzo-p-dioxin
TDO	tryptophan 2,3-dioxygenase;
TLR-4	Toll-like receptor
Trp	tryptophan
UbcH7	ubiquitin-conjugating enzyme H7
XA	xanthurenic acid
XRE, DRE	xenobiotic responsive element
